# Imaging study of coronal structural matching of the distal radius in normal adults

**DOI:** 10.1186/s12880-020-00434-x

**Published:** 2020-04-03

**Authors:** Xin Zhang, Feng Yuan, Yong Yin, Jian Fan

**Affiliations:** 1 Department of Orthopedics, Jiading District Central Hospital Affiliated Shanghai University of Medicine &Health Sciences, 1 Chengbei Road, Shanghai, 201800 China; 2 Department of Orthopedics, Tongji Hospital, School of Medicine,, Tongji University, 389 Xincun Road, Shanghai, 200065 China

**Keywords:** Distal radial fracture, Coronal position, Bone structure matching, Distal radioulnar joint

## Abstract

**Background:**

As an important anatomical basis, coronal structural position matching of the distal radius has long been lacking in terms of a quantitative understanding, and such matching is correlated with the postoperative functional recovery of patients with distal radius fracture. The purpose of this study was to explore the degree of coronal structural matching of the distal radius in a normal population and to improve the detailed anatomical knowledge of the distal radius.

**Methods:**

The reconstructed 3D data were analysed using 3-matic research software from thin-film CT images of 80 normal adults, and the coronal structural matching of the distal radius was studied from two aspects: 1) self-matching of the distal radius; and 2) matching between the distal radius and ulna (i.e., the joint space of the distal radioulnar joint). Specific research methods: 1) The relative position of the medial wall of the distal radius with respect to the lunate was determined as the percentage (%) of the vertical distance from the medial wall of the radius to the most prominent ulnar point of the lunate in the corresponding plane from the ulna to the radius. 2) A total of 9 sets of data were collected for evaluating the palmar lateral spacing, median spacing, and dorsal lateral spacing at the distal, middle, and proximal levels of the radius.

**Results:**

In the study, 9 sets of data were obtained. And the data of self-matching of the coronal structural of distal radius was also obtained, was 45.0% ± 16.2%. The *P* values in the above data were all greater than 0.05, showing no statistical significance. Finally, data of coronal bone structural matching of distal radius in 80 normal adults were obtained.

**Conclusions:**

Our study refines the anatomical data of the degree of coronal bone structural matching of the distal radius in a normal population. To explore the relationship between coronal alignment and function in cases of distal radius fracture, a standardized approach was established. Thin- film CT may help diagnose patients with dysplasia around the lunate and radioulnar joint that is difficult to diagnose on MRI.

## Background

Currently, in the study of distal radius fractures, the biomechanical and clinical correlations between the reduction of the bony structures (radial height, ulnar inclination, palmar inclination, and flatness of the carpal articular surface of the radius) and the resulting wrist function are relatively accurate [1]. However, in some patients with distal radius fracture, the reduction of the bony structure reaches the relevant standards, but the resulting clinical function is poor. We reviewed some literature and combined our clinical experience, concluded that coronal structural position matching of the distal radius may be an important factor affecting wrist function, which has not been paid much attention [2, 3]. In addition, the degree of coronal matching of the distal radius in the normal population should be urgently supplemented as important anatomical data. This study was divided into two parts: in one part, the coronal structural correlation between the distal radius and ulna was examined; in the other part, the degree of matching of the distal radius itself, which is related to the integrity of the medial wall of the distal radius, was examined. However, current related research is mainly limited to two dimensions. The majority of the understanding of the degree of coronal structural position matching of the distal radius is derived from the study of the bone structure of the distal radius and surrounding soft tissue in a pathological state after damage. Relevant research on the normal physiological state mainly relies on X-ray examination in the standard anterior-posterior projection, which not only causes data bias due to differences in the measurement position used by the researcher, thus preventing comparison of the data, but also limits the analysis to two dimensions. Meanwhile, surgeons are required to construct 3D morphological changes in their mind.

In this study, we focused on coronal structural matching of the distal radius in a normal adult population and first used 3D reconstruction techniques for quantitative analysis based on anatomical markers [4]. The study of the degree of matching of the distal radius on the coronal plane in normal adults can improve the understanding of the anatomy of the distal radius, improve the classification of distal radius fractures, and help orthopaedic surgeons make surgical plans before surgery. This work could deepen the understanding of distal radius fractures, be instructive for clinical work and provide data-based support for the design of new internal fixation devices.

## Methods

In this study, 80 normal adults with thin-slice CT images of the neutral wrist joint (40 males and 40 females) were randomly selected, and the corresponding parameters were recorded in detail. The slice thickness of thin-slice CT images is 0.625 mm. The data were imported into Mimics 20.0 software (Materialise, Belgium, 2017) to reconstruct the 3D surface model of the ulna and lunate. The reconstructed model was analysed using the 3-matic research software (Materialise, Belgium, 2017) associated with Mimics 20.0. All definitions and operations in this study were based on standardized coronal, sagittal, and transverse planes in 3D space, with the principal axis based on the central line of the medial wall of the distal radius.

Self-matching of the distal radius was based on the relative position of the medial wall of the distal radius with respect to the lunate and is expressed as the percentage (%) of the vertical distance from the medial wall of the radius to the most prominent ulnar point of the lunate in the corresponding plane from the ulna to the radius. Matching of the distal radius and ulna included 9 sets of data: the distal radioulnar joint was divided into the distal, middle and proximal levels, and data were obtained for the palmar line, median line and dorsal line at each level.

**Fig. 1:**
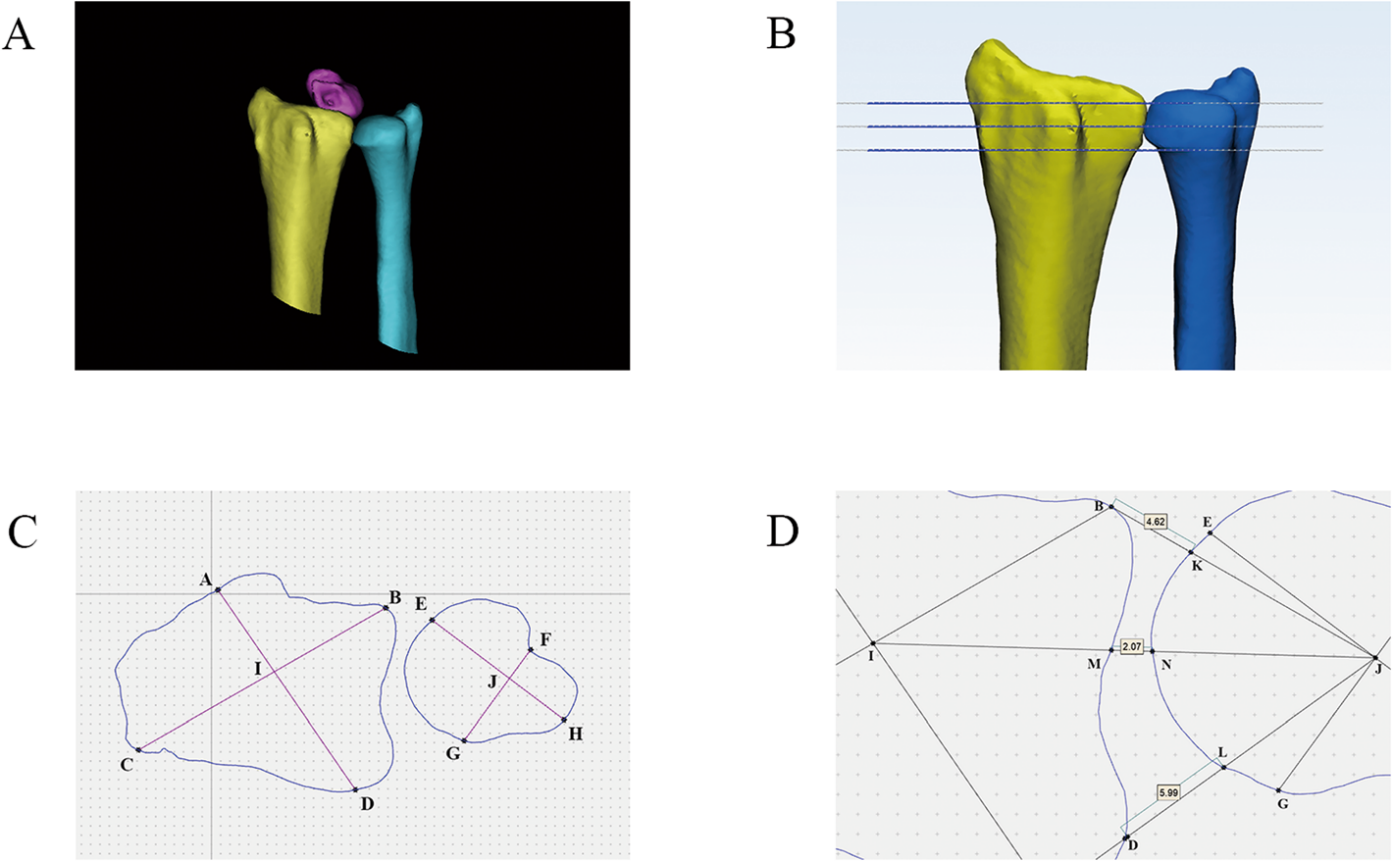
Schematic diagram of the process of measuring the wrist joint spacing. Fig. **a** shows the standard dorsal view of the ulna, radius and lunate after 3D reconstruction. Fig. **b** shows the distal, middle and proximal layers in the distal radioulnar joint. Fig. **c** shows the central point of the radial and ulnar contour projection map, taking the distal layer as an example. Fig. **d** shows the measurement of the palmar, median and dorsal lines

**Fig. 2:**
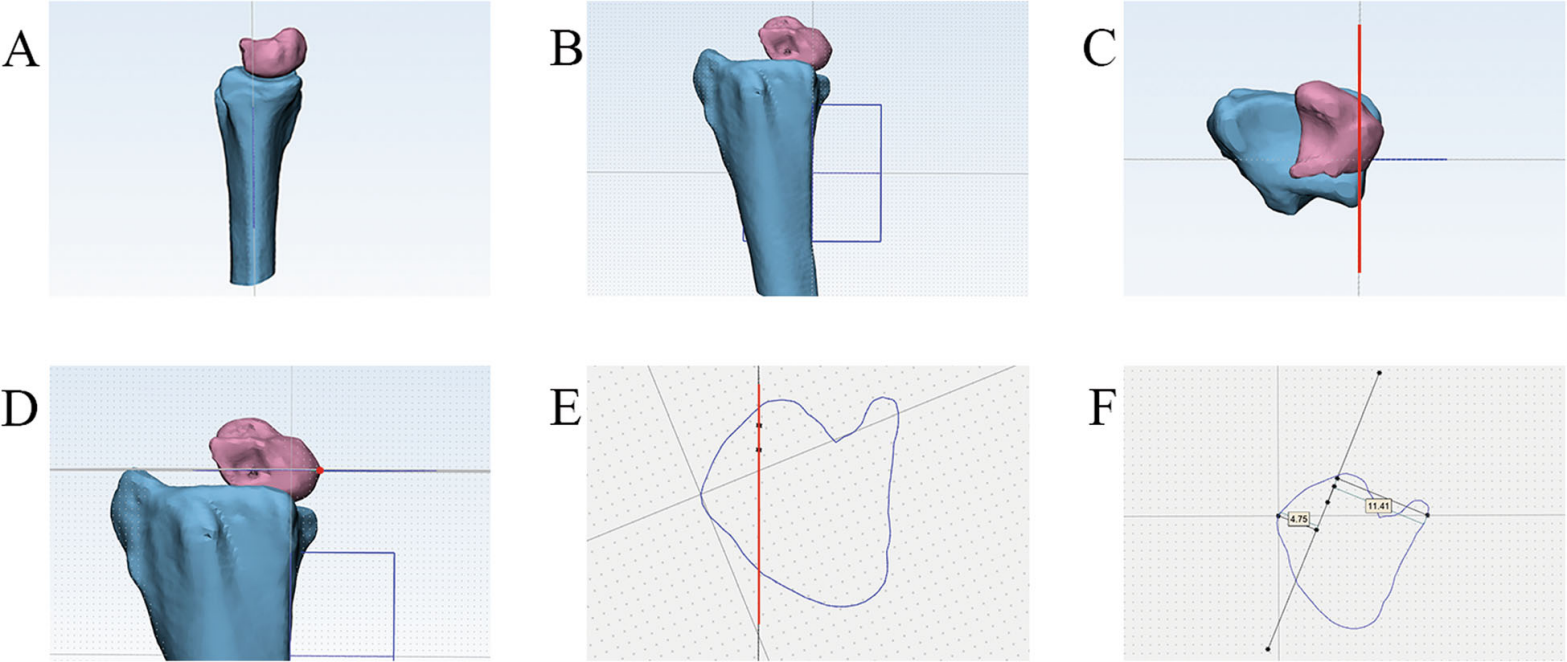
Schematic diagram of the measurement process in the coronal structural position matching of the distal radius. Fig. **a**-**c** maps the medial wall of the distal radius as the central axis. Fig. **d** maps a horizontal plane based on the most ulnar point of the lunate in the standard orthogonal position. Fig. **e** projects the outline of the lunate (blue line) and the medial wall of the radius (red line) on the horizontal plane through which the most ulnar point of the lunate passes. Fig. **f** maps the ulnar and radial protrusions of the lunate from the horizontal plane to the medial radius, resulting in a vertical distance

The specific operation process was as follows:

1. Examine normal adults with neutral thin-slice CT images of the wrist joints.
2. Record the relevant data of each subject in detail.
3. Reconstruct the radius, ulna, and lunate in Mimics 20.0 and distinguish them using different colours (Fig. 1a).
4. The reconstructed radius, ulna and lunate were imported into the 3-matic research software and matched using Mimics 20.0.
5. The relevant parameters were adjusted, and the final measurement results were set in units of “mm”.
6. After the lunate was hidden, the centre of the radioulnar joint was determined perpendicular to the radius and set to a basic plane, which was then translated to the proximal and distal 3 mm, respectively, and replicated, yielding three planes at the distal, middle and proximal levels (Fig. 1b).
7. Contour projection maps of the radius and ulna were obtained in the distal plane. Four points were identified on the contour maps of the radius (points A, B, C, and D) and ulna (points E, F, G, and H), respectively. The diagonal midpoint was used to determine the central point of the contour maps of the radius (point I) and ulna (point J) (Fig. 1c). When selecting points on the ulnar contour projection map, according to the anatomical position, reliable individual points were selected. The best location of the bony protuberance was within 0.5–1.0 cm of the joint.
8. Based on the centre point J of the ulna contour and points B, I and D of the radius contour, the intersection points of lines BJ and DJ with the ulna contour were defined as K and L, respectively, and the intersection points of line IJ with the radius and ulna contours were defined as M and N, respectively. Finally, the length of lines DL, MN and BK, i.e., the length of the palmar, median and dorsal lines, respectively, was measured (Fig. 1d).
9. Similarly, 9 sets of data were obtained on the distal, middle and proximal planes to quantitatively evaluate the distance between the ulna and radius.
10. The ulna was then hidden, and two planes perpendicular to each other were centred on the medial of the radius (Fig. 2a-c).
11. The coronal view of the radius was centred on the position of the lunate, passing through the most prominent point on the ulnar side of the lunate (Fig. 2d).
12. The shadow of the lunate and the 2 points on the inner side wall of the radius were projected on the horizontal plane (Fig. 2e).
13. The vertical distance from the most prominent ulnar point of the lunate to the medial wall of the radius was measured on the horizontal plane to quantitatively evaluate the degree of coronal matching of the distal end of the radius (Fig. 2f).

**Fig. 3:**
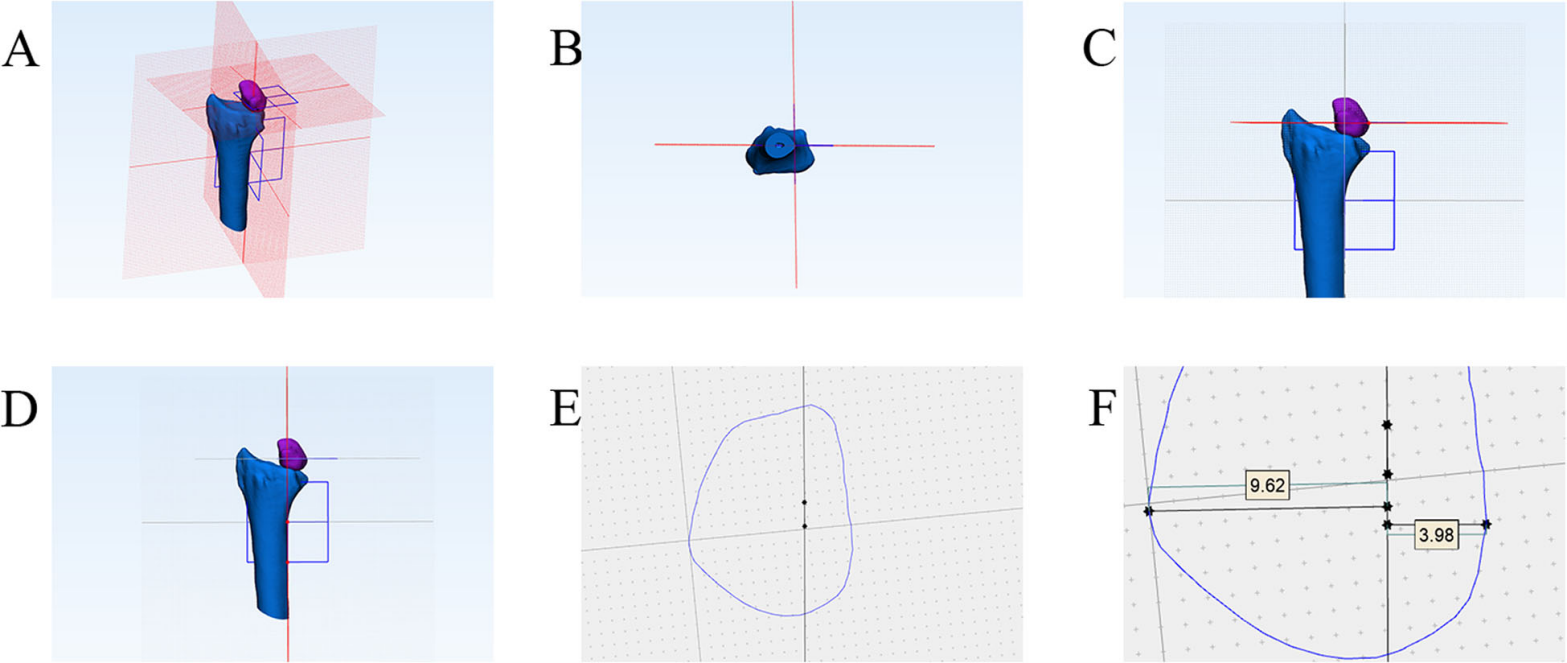
A case of the measurement process in the coronal structural position matching of the distal radius. Figure **a** shows the three planes of the coronal plane, the sagittal plane, and the mutually perpendicular cross sections centered on the medial wall of the distal radius. Figure **b** shows that the vertical axis of the medial wall of the distal radius, the intersection of the coronal and sagittal planes. Figure **c** shows a cross section through the point on the ulnar point of the lunate. Figure **d** shows 2 points on different sagittal planes of the medial wall of the distal radius. Figure **e** represents the projection of the above 2 points on the cross section, and the line represents the medial wall of the distal radius. Figure **f** shows the maximum vertical distance from the medial wall of the distal radius to the ulnar point(9.62 mm) and the radial point of the lunate (3.98 mm) in the lunar projection map. 9.62/(9.62 + 3.98)*100% = 70.7%, the 70.7% is the date of coronal structural position matching of the distal radius now

We used a set of pictures to illustrate the self-matching of the coronal structural of the distal radius (Fig. 3).

## Results

Data analysis was performed using SPSS 22.0 (IBM, Chicago, IL, USA) statistical software to analyse whether all sets of data were normally distributed. Statistical differences in the morphology based on the degree of coronal matching of the distal radius by sex were calculated using independent sample *t*-tests. Independent sample *t*-tests were used for comparisons between the sexes, and *X*^*2*^ tests were used for comparisons of different positions. Pairwise comparisons of spacing and the position of the medial wall of the distal radius relative to the lunate in the 9 sets were conducted by Pearson correlation analysis; *P* < 0.05 indicated statistically significant differences.

The subjects of this study were CT images normal, neutral wrist joints, and the analysis showed similar general indicators of coronal matching of the distal radius in normal adults of both sexes, namely, age and location; all *P* values were over 0.05, showing no statistical significance (Table 1). Nine sets of data were obtained, including the distal, middle, and proximal levels. The data of self-matching of the coronal structural of distal radius was also obtained. The *P* values of the above sets of data in both sexes were all greater than 0.05, showing no statistical significance (Table 2).

**Table 1:** Comparison of general data regarding the coronal position of the distal radius in normal adults of both sexes

Group	Age(^*−*^*X ± s*)	Side	
		Left	Right
Male(*n* = 40)	42.5 ± 15.3	21	19
Female(n = 40)	45.8 ± 12.6	17	23
*t*/*X*^*2*^	1.052	0.802	
*P*	0.296	0.370

**Table 2:** Coronal matching of the distal radius in normal adults of both sexes

	Spacing of Distal Radioulnar Joint (^−^*X ± s*)	Male(*n* = 40)	Female(*n* = 40)	*t*	*P*
Distal	Palmar Line	4.57 ± 1.78	4.61 ± 2.09	1.487	0.141
	Median Line	1.52 ± 0.95	1.82 ± 1.10	0.613	0.542
	Dorsal Line	2.63 ± 1.70	3.30 ± 1.60	0.787	0.434
Middle	Palmar Line	2.61 ± 1.51	2.81 ± 1.76	0.532	0.596
	Median Line	0.84 ± 0.53	0.91 ± 0.65	0.593	0.555
	Dorsal Line	2.42 ± 1.04	2.32 ± 1.20	0.403	0.688
Proximal	Palmar Line	4.57 ± 1.78	4.61 ± 2.09	0.090	0.928
	Median Line	1.52 ± 0.95	1.82 ± 1.02	1.351	0.180
	Dorsal Line	2.84 ± 1.71	3.22 ± 1.62	1.814	0.074
Self-Matching of Distal Radius		47.8% ± 17.2%	42.2% ± 14.9%	1.573	0.120

Note: unit, mm

Finally, we obtained data from 80 normal adults with coronal matching of the distal radius (Table 3). In the distal radioulnar joint, the spacing on the distal layer along the palmar line, median line, and dorsal line, was 4.57 ± 2.40 mm, 2.03 ± 1.07 mm, and 3.75 ± 1.49 mm, respectively. On the middle layer, the spacing along the palmar lateral line, median line and dorsal lateral line was 2.75 ± 1.65 mm, 0.88 ± 0.59 mm and 2.37 ± 1.12 mm, respectively. On the proximal layer, the spacing along the palmar lateral line, median line and dorsal lateral line was 4.59 ± 1.93 mm, 1.67 ± 0.99 mm and 3.03 ± 1.67 mm, respectively. The relative matching of the axis of the medial wall of the distal radius and the lunate was 45.0 ± 16.2%.

**Table 3:** Coronal matching of the distal radius in 80 normal adults

	Position	Spacing of Radioulnar Joint (mm)
Distal	Palmar Line	4.57 ± 2.40
	Median Line	2.03 ± 1.07
	Dorsal Line	3.75 ± 1.49
Middle	Palmar Line	2.71 ± 1.64
	Median Line	0.88 ± 0.59
	Dorsal Line	2.37 ± 1.12
Proximal	Palmar Line	4.59 ± 1.93
	Median Line	1.67 ± 0.99
	Dorsal Line	2.96 ± 1.67
Self-Matching of Distal Radius		45.0 ± 16.2%

Note: unit, mm

The *P* values in the 9 sets of data on the different layers and the relative matching of the medial wall of the coronal structural of the distal radius and the lunate were all greater than 0.05, showing no statistical significance. Pairwise comparisons were made among all sets of data measured in normal adults (Table 4).

**Table 4:** Pairwise correlation analysis of coronal matching of distal radius in normal adults

*P*		Distal			Middle			Proximal		
		Palmar Line	Median Line	Dorsal Line	Palmar Line	Median Line	Dorsal Line	Palmar Line	Median Line	Dorsal Line
Distal	Palmar Line	–	0	0.463	0	0.065	0.136	0.649	0.017	0.015
	Median Line	0	–	0	0.622	0.128	0.001	0.112	0.552	0.362
	Dorsal Line	0.463	0	–	0.233	0.123	0	0.972	0.941	0.118
Middle	Palmar Line	0	0.622	0.233	–	0.041	0.001	0.002	0.745	0.005
	Median Line	0.065	0.128	0.123	0.041	–	0.002	0.546	0	0
	Dorsal Line	0.136	0.001	0	0.001	0.002	–	0.422	0.025	0
Proximal	Palmar Line	0.649	0.112	0.972	0.002	0.546	0.422	–	0	0.901
	Median Line	0.017	0.552	0.941	0.745	0	0.025	0	–	0
	Dorsal Line	0.015	0.362	0.118	0.005	0	0	0.901	0	–
Self-Matching of Distal Radius		0.566	0.208	0.062	0.993	0.225	0.609	0.872	0.424	0.876

## Discussion

This study focused on the degree of matching of the coronal position of the distal radius in normal adults in the neutral position. Mark Ross et al. reported a method for judging the coronal structure of the distal radius on X-ray examination. The relative positions of the inner wall of the radius and the lunate demonstrated the reliability of this method for the first time, with a corresponding average degree of matching of 55% (26 to 75%) [5]. We applied thin-slice CT 3D reconstruction technology for the first time and determined that the position of the axis of the medial wall of the distal radius relative to the lunate was 45.0 ± 16.2% on the 3D reconstruction, which indicates more stable and consistent data, breaking through the limitations of the traditional imaging process. The distal radioulnar joint spacing was used as an important index to evaluate the matching of the distal radius with the ulna. For the first time, we used a total of 9 sets of data to perform a quantitative assessment at different levels. Although significant differences in the skeletal morphology of the distal radius have been reported between men and women [4], sex did not affect the variation in the radioulnar joint spacing. In the normal population, there was a correlation between the palmar line and the median line on each horizontal plane. And there was a correlation between the dorsal line and the median line on each horizontal plane (Table 4). But there were no correlations between the palmar lines and dorsal lines on the distal and proximal plane, not in Middle plane (Table 4). Those show that the distal radius as a spatial structure has its unique spatial law to be found. Once this rule is broken, it will indicate the existence of certain lesions such as fractures of the distal radius or injury of ligaments. This can provide theoretical guidance for the selection of wrist arthroscopy channels and diagnostic value for ligament injuries that are difficult to diagnose on MRI. Familiarity with the local anatomy and selection of the correct approach are critical in arthroscopic wrist surgery. Due to the narrow gap in the radioulnar joint, the importance of the surgical approach is particularly prominent. A radiocarpal tunnel can be established for a 360° view of the radiocarpal joint as needed. This study has important reference value in selecting the lens size and approach applied in operations involving the distal radioulnar joint.

The correlations of distal radioulnar joint spacing at the same position among the three levels were not significant, which is related to the anatomy of the capitulum ulnae. We believe that the thinner the horizontal section, the better the correlation. Among the 9 sets of measured data regarding the space between the ulna and radius, the narrowest space was along the median line on the middle layer, and the data showed the best consistency, with a value of 0.88 mm ± 0.59 mm. This is different from the clinical impression of the distal radioulnar joint spacing when the wrist is neutral in clinical work. On the one hand, it depends on the measurement method; on the other hand, it is related to partial rotation in the process of wrist radiography. Some scholars believe that the distal radioulnar joint space is an important part of coronal structural matching of the distal radius and that changes in the spacing will be reflected in changes in the relative position of the medial wall of the distal radius and the lunate [6, 7].

In this study, although the sigmoid notch of the radius was used as a distal extension of the osseous structure of the distal medial wall of the radius, there were no correlations between the position of the axis of the distal medial wall of the radius relative to the lunate and the length of any line at any level in the distal radioulnar joint. This means that there are two important indicators for exploring the degree of coronal structural matching of the distal radius: 1) the position of the medial wall of the distal radius relative to the lunate to evaluate the degree of structural matching of the radius itself; and 2) the spacing of the distal radioulnar joint to evaluate the coronal matching between the distal radius and ulna. Both of these are of great significance as independent indicators and have no absolute correlation. However, they cannot be completely separated in the research process and need to be analysed in a more specific way.

## Conclusion

Our study refines the anatomical data of the degree of coronal bone structural matching of the distal radius in a normal population. To explore the relationship between coronal alignment and function in cases of distal radius fracture, a standardized approach was established. Thin-slice CT may help diagnose patients with dysplasia around the lunate and radioulnar joint that is difficult to diagnose on MRI.

## Data Availability

The datasets used and/or analysed during the current study are available from the corresponding author on reasonable request.

## Abbreviations

CT: Computed Tomography
MRI: Magnetic Resonance Imaging
3D: three dimensional
